# Material exploration via designing spatial arrangement of octahedral units: a case study of lead halide perovskites

**DOI:** 10.1007/s12200-021-1227-z

**Published:** 2021-04-27

**Authors:** Pengfei Fu, Sanlue Hu, Jiang Tang, Zewen Xiao

**Affiliations:** 1 Wuhan National Laboratory for Optoelectronics, Huazhong University of Science and Technology, Wuhan, 430074 China; 2 School of Physics, Huazhong University of Science and Technology, Wuhan, 430074 China; 3 School of Optical and Electronic Information, Huazhong University of Science and Technology, Wuhan, 430074 China

**Keywords:** lead halide perovskite, electronic dimensionality, functional octahedral units, optoelectronic properties, photodetector

## Abstract

Halide perovskites have attracted tremendous attention as semiconducting materials for various optoelectronic applications. The functional metal-halide octahedral units and their spatial arrangements play a key role in the optoelectronic properties of these materials. At present, most of the efforts for material exploration focus on substituting the constituent elements of functional octahedral units, whereas designing the spatial arrangement of the functional units has received relatively little consideration. In this work, via a global structure search based on density functional theory (DFT), we discovered a metastable three-dimensional honeycomb-like perovskite structure with the functional octahedral units arranged through mixed edge- and corner-sharing. We experimentally confirmed that the honeycomb-like perovskite structure can be stabilized by divalent molecular cations with suitable size and shape, such as 2,2′-bisimidazole (BIM). DFT calculations and experimental characterizations revealed that the honeycomb-like perovskite with the formula of BIMPb_2_I_6_, synthesized through a solution process, exhibits high electronic dimensionality, a direct allowed bandgap of 2.1 eV, small effective masses for both electrons and holes, and high optical absorption coefficients, which indicates a significant potential for optoelectronic applications. The employed combination of DFT and experimental study provides an exemplary approach to explore prospective optoelectronic semiconductors via spatially arranging functional units.

**Electronic Supplementary Material:**

Supplementary material is available in the online version of this article at 10.1007/s12200-021-1227-z and is accessible for authorized users.

**Figure 1:**
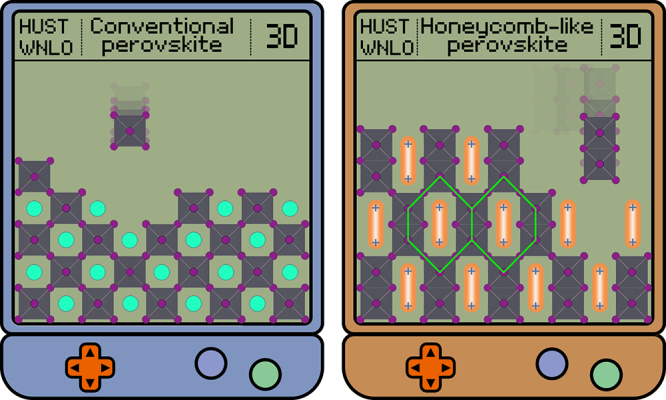

*These authors contributed equally.

## 1 Introduction

Halide perovskites, with the common formula $ABX_3$ — where A is an organic or alkali cation, B is commonly $Pb^{2+}$, and X is a halogen anion — have attracted tremendous attention as semiconducting materials not only for thin-film solar cells [1,2] but also for photodetectors [3–5] and light-emitting diodes (LEDs) [6–9] due to their superior optoelectronic properties [10–12] — such as suitable direct allowed bandgaps [13], small effective masses for both holes and electrons [14], high optical absorption coefficients [15], long photogenerated carrier diffusion lengths and lifetimes [16], and high defect tolerance [17–20] — as well as solution processability that enables low production costs. Structurally, these perovskites consist of a three-dimensional (3D) corner-sharing network of $[BX_6]$ octahedra (i.e., $BX_3$), with the "A" cations occupying 12-fold cuboctahedral voids within the network and counterbalancing the charge of $[BX_3]^-$ extended anion. The band-edge states, i.e., the upper valence and lower conduction bands, which play a key role in optoelectronic properties, are mainly derived from the 3D network of corner-sharing $[BX_6]$ octahedra. Therefore, the $[BX_6]$ octahedra can be the functional octahedral units [21] for these halide perovskite semiconductors. The high symmetry and connectivity of the $[BX_6]$ octahedra in these perovskites account for high electronic dimensionality [22], which is the primary enabler for the superior optoelectronic properties. Under certain conditions, some perovskites will convert to nonperovskite phases (often denoted as δ-$ABX_3$), which consist of either double chains of edge-sharing $[BX_6]$ octahedra, e.g., δ-$CsPbI_3$ and δ-$CsSnI_3$, or face-sharing $[BX_6]$ octahedra, e.g., δ-$HC(NH_2)_2PbI_3$ [23]. The lower symmetry and lower-dimensional connectivity for δ-$ABX_3$ lead to a lower electronic dimensionality, accompanied by large bandgaps, large effective masses, defect intolerance, and high anisotropy [12,24]. Moreover, with an increase in the precursor ratio of AX to $BX_2$, $ABX_3$ can be sliced into $A'_2A_{n-1}B_nX_{3n+1}$ (A′ is commonly a long organic molecular cation) with layers of corner-sharing [$BX_6$] octahedra ($A'_2BX_4$ for $n = 1$) [22,25,26], $A_3BX_5$ with single chains of corner-sharing [$BX_6$] octahedra [22,27], and $A_4BX_6$ with isolated [$BX_6$] octahedra [22,28,29]. The lowered electronic dimensionality with natural quantum confinement effect makes these reduced-dimensional perovskites a boon for luminescent materials [30–34]. In summary, not only the [$BX_6$] functional octahedral units but also their connectivity (in a certain spatial order) determine the optoelectronic properties of halide perovskites or nonperovskites.

Inspired by the success of lead iodide perovskites, substantial efforts have been made to search for various perovskite-based halide materials for optoelectronic applications [35,36]. At present, most efforts focus on material design by partially or completely substituting the constituent elements in the known perovskite structures, mainly the elements comprising the functional octahedral units, whereas relatively few efforts aim to expand the perovskite family by discovering structure motifs with designed arrangements of functional octahedral units. In this work, via a global structure search using $CsPbI_3$ as a paradigm, we discovered a metastable 3D honeycomb-like perovskite structure with the [$PbI_6$] functional octahedral units arranged by a mixed edge- and corner-sharing means. Experimentally, the honeycomb-like perovskite framework was stabilized by a divalent molecular cation with suitable size and shape, such as dications of 2,2′-bisimidazole (BIM). Density functional theory (DFT) calculations and experimental characterizations revealed that the honeycomb-like perovskite with the formula of $BIMPb_2I_6$, synthesized through a solution process, exhibited high electronic dimensionality, a direct allowed bandgap of 2.1 eV, small effective masses for both electrons and holes, and high optical absorption coefficients. A photodetector based on the $BIMPb_2I_6$ single crystal showed responsivity and photodetectivity of 0.1 mA/W and $1.1 \times 10^{10}$ Jones, respectively. This work provides an exemplary approach for designing prospective optoelectronic halide perovskites or perovskite derivatives by arranging the functional units.

## 2 Results and discussion

To unveil possible frameworks that perovskite-type compounds can form, we predicted energetically stable/metastable structures using $CsPbI_3$ as a paradigm. As shown in Fig. S1 in the Supporting Information, all the frameworks of the energetically stable/metastable structures comprised [$PbI_6$] functional octahedral units, which are arranged in different spatial orders. Already-synthesized δ-$CsPbI_3$ (orthorhombic, *Pnma*), γ-$CsPbI_3$ (orthorhombic, *Pnma*), and α-$CsPbI_3$ (cubic, $Pm\bar{3}m$) were involved in the predicted stable/metastable structures (Fig. 1(a)). Among them, δ-$CsPbI_3$, a nonperovskite phase that consists of low-dimensional chains of edge-sharing [$PbI_6$] octahedra, is the lowest-energy structure. Associated with low-dimensional structure, δ-$CsPbI_3$ exhibits a large bandgap (Fig. 1(b)) and large effective masses (Table S1), and thus is unsuitable for solar cells. The cubic perovskite phase α-$CsPbI_3$, which features as a 3D framework of corner-sharing [$PbI_6$] functional octahedral units, is a metastable structure with a formation energy of 30.3 meV/atom, which is higher than that of δ-$CsPbI_3$. Owing to the high symmetry and high electronic dimensionality, α-$CsPbI_3$ exhibits desired electronic properties for solar cells, including a suitable direct allowed bandgap, small effective masses for both electrons and holes, high optical absorption coefficients, and high defect tolerance. However, for use as a solar cell absorber, the intrinsically metastable α-$CsPbI_3$ could only be stabilized at room temperature in microscale or mesoscale using approaches, such as ligand passivation [37–40] and ion doping [41]. The orthorhombic γ-$CsPbI_3$, a distorted perovskite that is derived from α-$CsPbI_3$ via octahedral tilting [42], showed enhanced stability compared with the cubic α-$CsPbI_3$. The octahedral tilting slightly lowers the electronic dimensionality [22]. As a result, the bandgap and effective masses of the orthorhombic γ-$CsPbI_3$ are larger than those of the cubic α-$CsPbI_3$ (Table S1).

As shown in Fig. S1, most newly-predicted metastable structures are low-dimensional, including the face-sharing δ phase that is isostructural to δ-$HC(NH_2)_2PbI_3$ [23]. Interestingly, we found a 3D honeycomb-like perovskite structure, which holds the potential to provide a high electronic dimensionality for certain optoelectronic applications [22]. The honeycomb-like perovskite consists of

**Fig. 1** (a) Crystal structures, formation energies, and (b) band structures of newly-predicted honeycomb-like $CsPbI_3$ perovskite alone with already-synthesized α-, γ-, and δ-$CsPbI_3$ for comparison. The band structures are aligned by the averaged electrostatic potential, with the valence band maximum of α-$CsPbI_3$ set to zero pairs of edge-sharing $[PbI_6]$ octahedra (i.e., the $[Pb_2I_{10}]$ units) via conner-sharing, with Cs–Cs cation dimers (even slightly shorter than the Cs–Cs distance in elemental Cs) filled in flattened hexagonal hollow cells. Because of the reduced symmetry and thus reduced degeneracy, the honeycomb-like $CsPbI_3$ has a bandgap that is slightly larger than those for α-$CsPbI_3$ and γ-$CsPbI_3$ perovskites. Nevertheless, the honeycomb-like $CsPbI_3$ perovskite still exhibits desired electronic properties that are seen in lead halide perovskites, such as high electronic dimensionality, a direct allowed bandgap, small effective masses (Table S1), and high optical absorption coefficients, as shown in Fig. S2. However, like the α-$CsPbI_3$ and γ-$CsPbI_3$ perovskites, the honeycomb-like $CsPbI_3$ is a metastable structure with even higher formation energy (i.e., 9.0 meV/atom, higher than that of α-$CsPbI_3$) and should be easily converted to the δ-$CsPbI_3$ nonperovskite even if the honeycomb-like $CsPbI_3$ was successfully synthesized under certain conditions. We supposed substituting Cs–Cs dimer cations with divalent molecular cations with tailored size and shape could suppress phase conversion and stabilize the honeycomb-like perovskite structure, as illustrated in Fig. S3. Besides, to keep the desired high electronic dimensionality, the organic counterions should not cause significant structural distortion in the honeycomb-like perovskite or contribute to band edges with their localized molecular orbitals. Notably, most divalent molecular cations reported in the literature contribute to band edges of related compounds with localized molecular orbitals [43], whereas BIM is one of the few divalent molecular cations that do not contribute to the band edges [44], as confirmed by the DFT calculations (Figs. S4 and S5). Therefore, we focused on synthesizing the predicted 3D honeycomb-like perovskite based on this molecule, and further unveil their unique optoelectronic properties.

In this study, single crystals of $BIMPb_2I_6$ were grown by slowly cooling down a diluted HI solution containing stoichiometric BIMI and $PbI_2$. The stoichiometric ratio of Pb to I was confirmed to be 1:3 via the energy-dispersive X-ray spectroscopy (EDS) measurement, as shown in Fig. S6. Single-crystal X-ray diffraction (SCXRD) measurement revealed a monoclinic $C2/m$ system, with the detailed crystallographic data listed in Table S2. As shown in Fig. 2(a), the crystal structure of $BIMPb_2I_6$ resembles that of a honeycomb-like $CsPbI_3$, hence verified the previous expectation that the honeycomb-like perovskite structure could be stabilized by a suitable divalent molecule cation. Owing to the high symmetry, Pb atoms in the inorganic framework are equivalent, and iodine atoms have three different environments (Fig. S7). The bond length of three unique Pb−I bonds are 3.24, 3.20, and 3.15 Å, respectively (Table S3). For each $[PbI_6]$ octahedron in $BIMPb_2I_6$, angles of nonaxial I–Pb–I bonds range from 87° to 92° (Table S4). The corner-sharing Pb–I–Pb bonds are linear in the *ab*-plane and nearly linear (176.4°) along the *c*-axis. These results indicate that the 3D honeycomb-like inorganic framework exhibits negligible distortion with BIM molecule cations, which is highly desired for high electronic dimensionality, thus the framework has good carrier transport properties. The well-matched powder X-ray diffraction (PXRD) patterns (Fig. 2(b)) further substantiates the solved crystal structure as well as the high phase for the synthesized $BIMPb_2I_6$. Thermogravimetric analysis (Fig. S8) showed that the title compound decomposed at around 280°C, mainly attributed to the sublimation of the organic parts.

**Fig. 2** (a) Crystal structure of $BIMPb_2I_6$ viewed along the [001] direction; Pb, I, C, N, and H atoms are denoted by dark gray, purple, brown, light blue, and pink spheres, respectively. The octahedral nature of the Pb is illustrated using polyhedra. (b) Experimental and simulated powder X-ray diffraction (PXRD) patterns of $BIMPb_2I_6$. (c) Room-temperature low-frequency Raman spectra of $BIMPb_2I_6$. (d) Fourier transform infrared spectra of $BIMPb_2I_6$, along with the BIMI precursor for comparison

To gain insights into the dynamic behavior of the honeycomb-like perovskite framework, Raman measurement was performed at room temperature on a $BIMPb_2I_6$ crystal. Low-frequency Raman responses in lead halide perovskites are attributed to stretching or bending vibration modes of the Pb–I bonds [45–47]. For $BIMPb_2I_6$, as shown in Fig. 2(c), three peaks in the low-frequency range of 50–200 $cm^{-1}$ were found and well resolved. Indicated by the DFT results, shown in Fig. S9, the weak and strong peaks at 70 and 91 $cm^{-1}$, respectively, were attributed to the bending modes of edge-sharing Pb–I–Pb bonds, whereas the broad peak centered at 99 $cm^{-1}$ is attributed to the stretching modes of corner-sharing Pb–I–Pb bonds within the (*hk*0) planes. In addition, Fourier transform infrared (FT-IR) spectroscopy was recorded to reveal the interaction between organic molecules and inorganic framework. We found that the signal at around 3450 $cm^{-1}$ for $BIMPb_2I_6$ was significantly weaker than that of the $BIMI_2$ precursor, indicative of weak H-bonding interactions between the organic molecular cations and $[PbI_6]$ octahedral frameworks of $BIMPb_2I_6$ (Fig. 2(d)). The weak H-bonding interactions in $BIMPb_2I_6$ could also be understood from the decreased ∠N–H–I bonding angle (142°) in the structure model, which was due to the rigid inorganic framework and constrained orientation of organic molecules. (Fig. S10).

DFT calculations were performed for $BIMPb_2I_6$ with the experimentally determined crystal structure to understand the electronic properties before experimental characterizations. As shown in Fig. 3(a), $BIMPb_2I_6$ exhibits a direct bandgap of 2.10 eV at the M point. The valence band maximum (VBM) comprised antibonding states of the Pb 6s and I 5p orbitals, whereas the conductive band minimum (CBM) is derived mainly from the Pb 6p states (see Figs. 3(b) and S11). By contrast, the BIM molecular cation does not contribute to the band edges due to its relatively high electropositivity. Owing to the 3D connectivity of the band edge-determining $[PbI_6]$ octahedra, $BIMPb_2I_6$ exhibits a 3D electronic character. Associated with the high electronic dimensionality, the calculated band structure reveals relatively dispersive characters in all directions for the lower conduction and upper valence bands for $BIMPb_2I_6$. The calculated average effective masses for electrons and holes along the directions roughly parallel to the corner-sharing Pb–I–Pb bonds (i.e., the M–Y, M–Γ, and M–A directions) are $0.22m_0$ and $0.24m_0$, respectively (Table S1), indicating that both electrons and holes in $BIMPb_2I_6$ will be three-dimensionally mobile. Notably, the calculated effective masses for electrons and holes along M–V direction (parallel to edge-sharing octahedra) are $0.59m_0$ and $1.38m_0$, respectively. These values are relatively large but far superior to the near-infinite values for carrier transport across the layers of layered lead iodide perovskites with comparable bandgaps, such as $(CH_3NH_3)_2Pb(SCN)_2I_2$ [48]. The calculated sum of the squares of the dipole transition matrix elements ($P^2$) at various $k$ points revealed high transition probabilities between VBM and CBM. As a result, $BIMPb_2I_6$ exhibits high optical absorption coefficients in the order of $10^5$ $cm^{-1}$ (Fig. S12), as in the case with the typical corner-sharing lead halide perovskites.

**Fig. 3** (a) Calculated band structure of $BIMPb_2I_6$ along the $k$-path shown in the inset Brillouin zone. (b) Isosurface plots of charge density corresponding to conduction band minimum (CBM) and valence band maximum (VBM) at the M point. (c) Calculated transition matrix elements (unit: $Debye^2$) along the $k$-path shown in the inset Brillouin zone

Experimental characterizations were performed to further evaluate the electronic properties of $BIMPb_2I_6$. As shown in Fig. 4(a), the measured optical absorption spectrum of $BIMPb_2I_6$ showed an absorption onset at around 2 eV, which agreed well with the dark-red appearance of the crystals. It is known that the experimental optical absorption edges of metal halide perovskites are attributed to the fundamental band edges and the excitonic bands concurrently [49,50]. In this study, by using the Elliott model, with a Gaussian broadened exciton contribution, the Tauc plot for direct allowed transitions was fitted to yield a direct allowed bandgap of 2.1 eV and an exciton binding energy of ~40 meV (Fig. S13). This bandgap is larger than those for lead iodide perovskites but smaller than those for lead bromide perovskites. In addition, a steady-state photoluminescence (PL) spectrum was recorded for the powder sample of $BIMPb_2I_6$ under 400 nm excitation. As shown in Fig. 4(a), the PL spectrum showed a sharp and shoulder peak at 627 and around 656 nm, respectively. The sharp peak was ascribed to the exciton emissions. The shoulder peak may be assigned to the recombination of trap states, as indicated by the short PL lifetime (Fig. S14). Based on the calculated and

**Fig. 4** (a) Ultraviolet–visible (UV–Vis) absorption and photoluminance (PL) spectra of $BIMPb_2I_6$ powder. The inset depicts the dark-grown $BIMPb_2I_6$ crystals. (b) Current–voltage ($I$–$V$) curves of a detector based on a $BIMPb_2I_6$ single crystal under laser illumination ($\lambda$ = 637 nm) with different power intensities. (c) Switching cycles of photoresponse of the device under 20 $mW/cm^2$ of 637-nm laser at the bias voltage of 10 V. (d) A single switching cycle of photoresponse. The rise time ($t_r$) and fall time ($t_f$) are intervals that photocurrent rise from 10% to 90% and fall from 90% to 10%, respectively characterized electronic properties, we supposed that $BIMPb_2I_6$ could be a prospective semiconductor for photodetection. Therefore, a photodetector based on a single crystal of $BIMPb_2I_6$ has been fabricated with a planar electrode configuration. As shown in Fig. 4(b), the current–voltage (*I–V*) curves exhibited a typical Ohmic contact feature with a low dark current (0.28 nA @ 10 V bias). In addition, the photocurrent increased to 1.3 nA under 5 $mW/cm^2$ illumination with the same bias, which means the $BIMPb_2I_6$-based photodetector showed a clear photoresponse under a 637 nm light source. The current increased with the enhancement of the power density of the light source and finally saturated under ~30 $mW/cm^2$. As essential parameters to evaluate the performance of a photoconductor, the responsivity (*R*) and the specific potodetectivity ($D^*$) of our device were estimated to be ~0.10 mA/W and $1.1 \times 10^{10}$ Jones, respectively. To test the device stability, time-dependent photoresponse was measured under 20 $mW/cm^2$ of 637-nm irradiation controlled by switching on/off cycles at a bias voltage of 10 V. As shown in Fig. 4(c), uniform and repeatable photocurrent responses were observed for each switch-on/off cycle. The rising time ($t_r$) and fall time ($t_f$) were determined to be 1.0 and 0.8 ms, respectively (Fig. 4(d)). These preliminary results indicated that honeycomb-like lead halide perovskites represented by $BIMPb_2I_6$ could be prospective candidates for highly efficient photodetector materials. Therefore, $BIMPb_2I_6$ deserves further experimental optimization.

## 3 Conclusions

In summary, we discovered a honeycomb-like perovskite structure that preserves 3D electronic dimensionality by designing the spatial arrangement of the functional octahedral units. Experimentally, the target structure was achieved using a screened divalent molecular cation BIM as a stabilizer. DFT calculations indicated that the synthesized $BIMPb_2I_6$ honeycomb-like perovskite possesses high electronic dimensionality and desired optoelectronic properties such as a direct allowed bandgap, small carrier effective masses, and high optical absorption coefficients, whereas experimental characterizations revealed that it exhibited a moderate bandgap of 2.1 eV and PL at 627 nm. The photodetectors fabricated on the basis of $BIMPb_2I_6$ single crystals showed responsivity and photodetectivity of 0.1 mA/W and $1.1 \times 10^{10}$ Jones, respectively. The results provided an exemplary approach for designing prospective optoelectric semiconductors via spatially arranging functional units.

## 4 Experimental section

*DFT calculation*: DFT calculations were performed using the projector-augmented wave method as implemented in the Vienna *Ab initio* Simulation Package (VASP) 5.4 code [51]. The plane-wave cutoff energy was set to 500 eV. The search of crystal structure for $CsPbI_3$ was conducted using the particle swarm optimization method as implemented in the Crystal Structure Analysis by Particle Swarm Optimization (CALYPSO) code [52,53]. The generalized gradient approximation (GGA) Perdew–Burke–Ernzerhof (PBE) [54] was used as the exchange-correlation functional for structural relaxation and total-energy calculations. Γ-centered *k*-meshes with *k*-spacing of 0.2 $Å^{-1}$ were employed for sampling the Brillouin zones. All crystal structures were fully relaxed until the total force on each atom was $< 0.01$ eV/Å. The vibrational frequencies were obtained using the finite displacement method as implemented in the Phonopy code [55], and the Raman activity was then estimated by calculating the polarizability of the vibrational models using the VASP_RAMAN.PY code [56]. The calculations of electronic properties were performed using the Heyd–Scuseria–Ernzerhof (HSE) [57,58] hybrid functionals with the mixing parameter of 39% and the inclusion of spin–orbit coupling, which provided good bandgap descriptions for γ-$CsPbI_3$ and $BIMPb_2I_6$. The transition matrix elements were calculated using the VASP_TDM code [59].

*Material synthesis*: $PbI_2$ (AR, 99.9%), hydroiodic acid (AR, 45–50 wt.% in water, stabilized by 1.5% $H_3PO_2$), and hypophosphorous acid (AR, 50 wt.% solution in water) were purchased from Aladdin. All reagents were used as received without further purification. BIM was synthesized with the methodology described in Ref. [60]. The final product was recrystallized in concentrated HI to afford the protonated product, $BIMI_2$. An amount of 1 mmol (461 mg) $PbI_2$ was dissolved in 9-mL HI solution under 120°C to obtain a clear yellow solution, and 2-mL $H_2O$ was then added to dilute the solution. An amount of 0.5 mmol (148 mg) $BIMI_2$ was dissolved in 1-mL hypophosphorous acid in another vial. The solution was slowly added to the previous solution under heating. The temperature was lowered to 60°C until dark-red crystals precipitated as the title compound.

*Material structure determination*: The elemental analysis was conducted using a field-emission scanning electron microscope (FEI Nova NanoSEM 450) equipped with an EDS. A crystal sample with a suitable size was selected for SCXRD on Bruker D8 VENTURE diffractometer, using Mo Kα radiation ($\lambda = 0.71073$ Å) (50 kV, 30 mA) at 150 K. The collected data were integrated and applied with multiscan absorption corrections using the APEX-III software. The crystal structure was solved and refined by the SHLEXT (intrinsic phasing method) and SHLEXL (full-matrix least-squares on $F^2$) programs, respectively. The VESTA program [61] was used for structure visualization. The Crystallographic Information File (CIF) can be obtained from the Cambridge Crystallographic Data Centre (CCDC) by quoting the deposition number: CCDC 2074690. PXRD measurement was conducted on Brucker D8AA25 diffractometer with Cu Kα radiation ($\lambda$ = 1.54178 Å). The diffractograms were recorded in the range of $2\theta = 5°$ to 65° at 0.02° per step, whereas the integrated time of each step is 0.4 s. Simulated powder diffraction patterns were calculated using the solved crystal structure with VESTA.

*Material property characterization*: Raman spectra were acquired on LabRAM HR800 using a 732-nm laser as the excitation source. The spectra were collected from 50 to 200 $cm^{-1}$. FT-IR spectra were recorded on Bruker VERTEX 70 equipped with an attenuated total reflection accessory. The attenuated signals of solid samples were recorded and solved by FT in the mid-IR ranges (500–4000 $cm^{-1}$). Optical absorption spectroscopy measurements were performed on a Shimadzu 2600i UV–Vis spectrometer. The optical diffuse reflectance was measured in an integrated sphere using $BaSO_4$ powder as reference. The steady-states PL spectrum was measured on a Horiba-FL3 spectrofluorometer with a 400-nm excitation source. A 425-nm spectra LED was used as the excitation source of time-resolved PL. The emission counts were monitored at the maximum of the emission curve. The lifetime was obtained and calculated via the third-exponential fitting. Thermal stability was estimated by thermogravimetric coupled with differential thermal analysis performed on PerkinElmer Diamond TG/DTA. The powder samples were heated from room temperature to 500°C with a heating rate of 10°C/min. All measurements were conducted under an $N_2$ flow atmosphere.

*Photoresponse characterization*: Photoelectric measurements were performed using a planar electrode configuration. The electrode materials were proven not to have any obvious influence on the photoelectric properties. The current–voltage (*I*–*V*) and photocurrent–time (*I*–*t*) with the light on or off (measured at zero bias) were measured using a high-precision electrometer (Keithley 6517B). A THORLABS 637-nm pigtailed laser diode (LP637-MF300) was used for Vis light illumination. The incident light intensity was measured via a light power meter. The temperature during measurements was kept at 298 K using a Linkam TS1500 heating stage.

The responsivity ($R$) and photodetectivity ($D^*$) of $BIMPb_2I_6$ were determined, respectively, as follows:

$$R = J_{\mathrm{ph}}/L_{\mathrm{light}},$$

$$D^* = J_{\mathrm{ph}}/[L_{\mathrm{light}} \times (2eJ_{\mathrm{d}})^{1/2}],$$

where $J_{\mathrm{ph}}$, $L_{\mathrm{light}}$, and $J_{\mathrm{d}}$ represent photocurrent, incident light intensity, and dark current, respectively, and the constant $e$ is the elementary charge ($1.6 \times 10^{-19}$ C).

**Acknowledgements** This work was financially supported by the National Natural Science Foundation of China (NSFC) (Grant No. 51972130), Startup Fund of Huazhong University of Science and Technology, and Director Fund of Wuhan National Laboratory for Optoelectronics. The authors thank Prof. Kezhao Du in Fujian Normal University, China for the UV–Vis measurement, Yunpeng Yao in Fujian Institute of Research on the Structure of Matter, Chinese Academy of Sciences, China for providing experimental assistance toward photoresponse measurement, and Prof. Zhijun Ning in ShanghaiTech University, China for supporting the early-stage exploration.

**Electronic Supplementary Material** Supplementary material is available in the online version of this article at https://doi.org/10.1007/s12200-021-1227-z and is accessible for authorized users.

**Open Access** This article is licensed under a Creative Commons Attribution 4.0 International License, which permits use, sharing, adaptation, distribution and reproduction in any medium or format, as long as you give appropriate credit to the original author(s) and the source, provide a link to the Creative Commons licence, and indicate if changes were made. The images or other third party material in this article are included in the article's Creative Commons licence, unless indicated otherwise in a credit line to the material. If material is not included in the article's Creative Commons licence and your intended use is not permitted by statutory regulation or exceeds the permitted use, you will need to obtain permission directly from the copyright holder.

To view a copy of this licence, visit http://creativecommons.org/licenses/by/4.0/.

## Supporting Information

Material exploration via designing the spatial arrangement of functional octahedral units: a case study of lead halide perovskites

## References

[1] Kim J Y, Lee J W, Jung H S, Shin H, Park N G (2020). High-efficiency perovskite solar cells. Chemical Reviews.

[2] Jena A K, Kulkarni A, Miyasaka T (2019). Halide perovskite photovoltaics: background, status, and future prospects. Chemical Reviews.

[3] Yu X, Tsao H N, Zhang Z, Gao P (2020). Miscellaneous and perspicacious: hybrid halide perovskite materials based photodetectors and sensors. Advanced Optical Materials.

[4] Fang Y, Dong Q, Shao Y, Yuan Y, Huang J (2015). Highly narrowband perovskite single-crystal photodetectors enabled by surface-charge recombination. Nature Photonics.

[5] Wang H P, Li S, Liu X, Shi Z, Fang X, He J H (2021). Low-dimensional metal halide perovskite photodetectors. Advanced Materials.

[6] Tan Z K, Moghaddam R S, Lai M L, Docampo P, Higler R, Deschler F, Price M, Sadhanala A, Pazos L M, Credgington D, Hanusch F, Bein T, Snaith H J, Friend R H (2014). Bright light-emitting diodes based on organometal halide perovskite. Nature Nanotechnology.

[7] Xu W, Hu Q, Bai S, Bao C, Miao Y, Yuan Z, Borzda T, Barker A J, Tyukalova E, Hu Z, Kawecki M, Wang H, Yan Z, Liu X, Shi X, Uvdal K, Fahlman M, Zhang W, Duchamp M, Liu J M, Petrozza A, Wang J, Liu L M, Huang W, Gao F (2019). Rational molecular passivation for high-performance perovskite light-emitting diodes. Nature Photonics.

[8] Luo J, Wang X, Li S, Liu J, Guo Y, Niu G, Yao L, Fu Y, Gao L, Dong Q, Zhao C, Leng M, Ma F, Liang W, Wang L, Jin S, Han J, Zhang L, Etheridge J, Wang J, Yan Y, Sargent E H, Tang J (2018). Efficient and stable emission of warm-white light from lead-free halide double perovskites. Nature.

[9] Yan C, Lin K, Lu J, Wei Z (2020). Composition engineering to obtain efficient hybrid perovskite light-emitting diodes. Frontiers of Optoelectronics.

[10] Huang J, Yuan Y, Shao Y, Yan Y (2017). Understanding the physical properties of hybrid perovskites for photovoltaic applications. Nature Reviews. Materials.

[11] Xiao Z, Yan Y (2017). Progress in theoretical study of metal halide perovskite solar cell materials. Energy Materials.

[12] Yin W J, Shi T, Yan Y (2014). Unique properties of halide perovskites as possible origins of the superior solar cell performance. Advanced Materials.

[13] Meng W, Wang X, Xiao Z, Wang J, Mitzi D B, Yan Y (2017). Parity-forbidden transitions and their impact on the optical absorption properties of lead-free metal halide perovskites and double perovskites. Journal of Physical Chemistry Letters.

[14] Umari P, Mosconi E, De Angelis F (2014). Relativistic GW calculations on CH_3_NH_3_PbI_3_ and CH_3_NH_3_SnI_3_ perovskites for solar cell applications. Scientific Reports.

[15] Green M A, Jiang Y, Soufiani A M, Ho-Baillie A (2015). Optical properties of photovoltaic organic-inorganic lead halide perovskites. Journal of Physical Chemistry Letters.

[16] Stranks S D, Eperon G E, Grancini G, Menelaou C, Alcocer M J P, Leijtens T, Herz L M, Petrozza A, Snaith H J (2013). Electron-hole diffusion lengths exceeding 1 micrometer in an organometal trihalide perovskite absorber. Science.

[17] Yin W J, Shi T, Yan Y (2014). Unusual defect physics in CH_3_NH_3_PbI_3_ perovskite solar cell absorber. Applied Physics Letters.

[18] Kang J, Wang L W (2017). High defect tolerance in lead halide perovskite CsPbBr_3_. Journal of Physical Chemistry Letters.

[19] Huang H, Bodnarchuk M I, Kershaw S V, Kovalenko M V, Rogach A L (2017). Lead halide perovskite nanocrystals in the research spotlight: stability and defect tolerance. ACS Energy Letters.

[20] Meggiolaro D, Motti S G, Mosconi E, Barker A J, Ball J, Andrea Riccardo Perini C, Deschler F, Petrozza A, De Angelis F (2018). Iodine chemistry determines the defect tolerance of lead-halide perovskites. Energy & Environmental Science.

[21] Chen K, Li L (2019). Ordered structures with functional units as a paradigm of material design. Advanced Materials.

[22] Xiao Z, Meng W, Wang J, Mitzi D B, Yan Y (2017). Searching for promising new perovskite-based photovoltaic absorbers: the importance of electronic dimensionality. Materials Horizons.

[23] Stoumpos C C, Malliakas C D, Kanatzidis M G (2013). Semiconducting tin and lead iodide perovskites with organic cations: phase transitions, high mobilities, and near-infrared photoluminescent properties. Inorganic Chemistry.

[24] Brgoch J, Lehner A J, Chabinyc M L, Seshadri R (2014). Ab initio calculations of band gaps and absolute band positions of polymorphs of RbPbI_3_ and CsPbI_3_: implications for main-group halide perovskite photovoltaics. Journal of Physical Chemistry C.

[25] Saparov B, Mitzi D B (2016). Organic-inorganic perovskites: structural versatility for functional materials design. Chemical Reviews.

[26] Kahwagi R F, Thornton S T, Smith B, Koleilat G I (2020). Dimensionality engineering of metal halide perovskites. Frontiers of Optoelectronics.

[27] Raptopoulou C P, Terzis A, Mousdis G A, Papavassiliou G C (2002). Preparation, structure and optical properties of [CH_3_SC (NH_2_)_2_]_3_SnI_5_, [CH_3_SC(NH_2_)_2_][HSC(NH_2_)_2_]SnBr_4_, (CH_3_C_5_H_4_NCH_3_)PbBr_3_, and [C_6_H_5_CH_2_SC(NH_2_)_2_]_4_Pb_3_I_10_. Zeitschrift für Naturforschung. Teil B. Anorganische Chemie, Organische Chemie, Biochemie, Biophysik, Biologie.

[28] Saidaminov M I, Almutlaq J, Sarmah S, Dursun I, Zhumekenov A A, Begum R, Pan J, Cho N, Mohammed O F, Bakr O M (2016). Pure Cs_4_PbBr_6_: highly luminescent zero-dimensional perovskite solids. ACS Energy Letters.

[29] Zhang Y, Saidaminov M I, Dursun I, Yang H, Murali B, Alarousu E, Yengel E, Alshankiti B A, Bakr O M, Mohammed O F (2017). Zero-dimensional Cs_4_PbBr_6_ perovskite nanocrystals. Journal of Physical Chemistry Letters.

[30] Lin H, Zhou C, Tian Y, Siegrist T, Ma B (2018). Low-dimensional organometal halide perovskites. ACS Energy Letters.

[31] Smith M D, Connor B A, Karunadasa H I (2019). Tuning the luminescence of layered halide perovskites. Chemical Reviews.

[32] Tan Z, Li J, Zhang C, Li Z, Hu Q, Xiao Z, Kamiya T, Hosono H, Niu G, Lifshitz E, Cheng Y, Tang J (2018). Highly efficient blue-emitting Bidoped Cs_2_SnCl_6_ perovskite variant: photoluminescence induced by impurity doping. Advanced Functional Materials.

[33] Li J, Tan Z, Hu M, Chen C, Luo J, Li S, Gao L, Xiao Z, Niu G, Tang J (2019). Antimony doped Cs_2_SnCl_6_ with bright and stable emission. Frontiers of Optoelectronics.

[34] Tan Z, Chu Y, Chen J, Li J, Ji G, Niu G, Gao L, Xiao Z, Tang J (2020). Lead-free perovskite variant solid solutions Cs_2_Sn_1__*x*_Te_*x*_Cl_6_: bright luminescence and high anti-water stability. Advanced Materials.

[35] Xiao Z, Song Z, Yan Y (2019). From lead halide perovskites to lead-free metal halide perovskites and perovskite derivatives. Advanced Materials.

[36] Zhao X G G, Yang D, Ren J C C, Sun Y, Xiao Z, Zhang L (2018). Rational design of halide double perovskites for optoelectronic applications. Joule.

[37] Swarnkar A, Marshall A R, Sanehira E M, Chernomordik B D, Moore D T, Christians J A, Chakrabarti T, Luther J M (2016). Quantum dot-induced phase stabilization of α-CsPbI_3_ perovskite for high-efficiency photovoltaics. Science.

[38] Jiang Y, Yuan J, Ni Y, Yang J, Wang Y, Jiu T, Yuan M, Chen J (2018). Reduced-dimensional α-CsPbX_3_ perovskites for efficient and stable photovoltaics. Joule.

[39] Han B, Cai B, Shan Q, Song J, Li J, Zhang F, Chen J, Fang T, Ji Q, Xu X, Zeng H (2018). Stable, efficient red perovskite light-emitting diodes by (α,δ)-CsPbI_3_ phase engineering. Advanced Functional Materials.

[40] Chen K, Zhong Q, Chen W, Sang B, Wang Y, Yang T, Liu Y, Zhang Y, Zhang H (2019). Short-chain ligand-passivated stable α-CsPbI_3_ quantum dot for all-inorganic perovskite solar cells. Advanced Functional Materials.

[41] Hu Y, Bai F, Liu X, Ji Q, Miao X, Qiu T, Zhang S (2017). Bismuth incorporation stabilized α-CsPbI_3_ for fully inorganic perovskite solar cells. ACS Energy Letters.

[42] Woodward P M (1997). Octahedral tilting in perovskites. I. Geometrical considerations. Acta Crystallographica. Section B, Structural Science.

[43] Umeyama D, Leppert L, Connor B A, Manumpil M A, Neaton J B, Karunadasa H I (2020). Expanded analogs of three-dimensional lead-halide hybrid perovskites. Angewandte Chemie International Edition.

[44] Tang Z, Guan J, Guloy A M (2001). Synthesis and crystal structure of new organic-based layered perovskites with 2,2′-biimidazolium cations. Journal of Materials Chemistry.

[45] Quarti C, Grancini G, Mosconi E, Bruno P, Ball J M, Lee M M, Snaith H J, Petrozza A, Angelis F D (2014). The Raman spectrum of the CH_3_NH_3_PbI_3_ hybrid perovskite: interplay of theory and experiment. Journal of Physical Chemistry Letters.

[46] Cortecchia D, Neutzner S, Srimath Kandada A R, Mosconi E, Meggiolaro D, De Angelis F, Soci C, Petrozza A (2017). Broadband emission in two-dimensional hybrid perovskites: the role of structural deformation. Journal of the American Chemical Society.

[47] Yaffe O, Guo Y, Tan L Z, Egger D A, Hull T, Stoumpos C C, Zheng F, Heinz T F, Kronik L, Kanatzidis M G, Owen J S, Rappe A M, Pimenta M A, Brus L E (2017). Local polar fluctuations in lead halide perovskite crystals. Physical Review Letters.

[48] Xiao Z, Meng W, Saparov B, Duan H S, Wang C, Feng C, Liao W, Ke W, Zhao D, Wang J, Mitzi D B, Yan Y (2016). Photovoltaic properties of two-dimensional (CH_3_NH_3_)_2_Pb(SCN)_2_I_2_ perovskite: a combined experimental and density functional theory study. Journal of Physical Chemistry Letters.

[49] Saba M, Cadelano M, Marongiu D, Chen F, Sarritzu V, Sestu N, Figus C, Aresti M, Piras R, Lehmann A G, Cannas C, Musinu A, Quochi F, Mura A, Bongiovanni G (2014). Correlated electron-hole plasma in organometal perovskites. Nature Communications.

[50] Shi J, Zhang H, Li Y, Jasieniak J J, Li Y, Wu H, Luo Y, Li D, Meng Q (2018). Identification of high-temperature exciton states and their phase-dependent trapping behaviour in lead halide perovskites. Energy & Environmental Science.

[51] Kresse G, Furthmüller J (1996). Efficient iterative schemes for *ab initio* total-energy calculations using a plane-wave basis set. Physical Review B: Condensed Matter.

[52] Wang Y, Lv J, Zhu L, Ma Y (2010). Crystal structure prediction via particle-swarm optimization. Physical Review B: Condensed Matter and Materials Physics.

[53] Wang Y, Lv J, Zhu L, Ma Y (2012). CALYPSO: a method for crystal structure prediction. Computer Physics Communications.

[54] Perdew J P, Burke K, Ernzerhof M (1996). Generalized gradient approximation made simple. Physical Review Letters.

[55] Togo A, Tanaka I (2015). First principles phonon calculations in materials science. Scripta Materialia.

[56] Zheng Q. VASP_TDM, https://github.com/QijingZheng/VASP_TDM, 2016

[57] Heyd J, Scuseria G E, Ernzerhof M (2003). Hybrid functionals based on a screened Coulomb potential. Journal of Chemical Physics.

[58] Heyd J, Scuseria G E, Ernzerhof M (2006). Erratum: “Hybrid functionals based on a screened Coulomb potential”. Journal of Chemical Physics.

[59] Fonari A, Stauffer S. VASP_RAMAN.PY, https://github.com/raman-sc/VASP, 2013

[60] Matsumoto S, Watanabe M, Akazome M (2018). Incrementing Stokes shifts through the formation of 2,2′-biimidazoldiium salts. Organic Letters.

[61] Momma K, Izumi F (2008). VESTA: a three-dimensional visualization system for electronic and structural analysis. Journal of Applied Crystallography.

