# Supplementary material for: Material exploration via designing spatial arrangement of octahedral units: a case study of lead halide perovskites
Source: Front Optoelectron. 2021 Apr 27;14(2):252–9. doi: 10.1007/s12200-021-1227-z (PMC9743903; doi:10.1007/s12200-021-1227-z)
Supplement: Supplementary file 1 — Material exploration via designing the spatial arrangement of functional octahedral units: a case study of lead halide perovskites [file 12200_2021_1227_MOESM1_ESM.pdf]

Supporting Information

Material exploration via designing the spatial arrangement of functional octahedral units: a case study of lead halide perovskites

Pengfei Fu, Sanlue Hu, Jiang Tang, and Zewen Xiao

Table S1 Calculated effective masses for electrons and holes in δ-$CsPbI_3$, γ-$CsPbI_3$, α-$CsPbI_3$, honeycomb-like $CsPbI_3$, and honeycomb-like $BIMPb_2I_6$

		m_e*/m_0	m_h*/m_0
δ-$CsPbI_3$	X–Γ	0.98	1.09
	X–S	0.29	1.24
γ-$CsPbI_3$	Γ–Y	0.24	0.27
	Γ–Z	0.23	0.27
α-$CsPbI_3$	R–X	0.14	0.16
	R–M	0.18	0.20
honeycomb-like $CsPbI_3$	M–Y	0.21	0.17
	M–V	0.46	7.57
	M–Γ	0.22	0.22
	M–A	0.28	0.24
honeycomb-like $BIMPb_2I_6$	M–Y	0.20	0.21
	M–V	0.59	1.38
	M–Γ	0.22	0.26
	M–A	0.23	0.26

Table S2 Crystallographic information of $BIMPb_2I_6$

compound	$BIMPb_2I_6$
empirical formula	$C_6H_8N_4Pb_2I_6$
formula weight	1311.94
temperature/K	150.0
crystal system	monoclinic
space group	$C2/m$
a/Å	9.0913(3)
b/Å	17.9755(6)
c/Å	6.4049(2)
α/(°)	90
β/(°)	95.9020(10)
γ/(°)	90
Volume/Å^3	1041.14(6)
Z	2
ρ_{calc}/(g·cm^{-3})	4.185
μ/mm^{-1}	25.036
F(000)	1108.0
2Θ range for data collection/(°)	4.532 to 50.048
index ranges	$-10 \le h \le 10$, $-21 \le k \le 21$, $-7 \le l \le 7$
reflections collected	12090
independent reflections	951 [R_{int} = 0.0327, R_{sigma} = 0.0136]
data/restraints/parameters	951/0/47
goodness-of-fit on F^2	1.299
final R indexes [I>=2σ(I)]	R_1 = 0.0214, wR_2 = 0.0615
final R indexes [all data]	R_1 = 0.0216, wR_2 = 0.0616
largest diff. peak/hole/(e·Å^{-3})	2.92/–1.62

Table S3 Selected bond lengths of $BIMPb_2I_6$

label (atom−atom)	length/Å
Pb(1)—I(1)	3.2379(4)
Pb(1) − I(1)^1	3.2379(4)
Pb(1) − I(2)	3.20403(10)
Pb(1) − I(2)^2	3.20403(10)
Pb(1) − I(3)	3.15016(19)
Pb(1) − I(3)^3	3.15016(19)

^{1}1–X, 1–Y, 2–Z; ^{2}X, Y, 1+Z; ^{3}1–X, Y, 2–Z

Table S4 Selected bond angles of $BIMPb_2I_6$

label (atom−atom−atom)	angle/(°)
I(1)^{1} − Pb(1) − I(1)	88.839(15)
I(2) − Pb(1) − I(1)	89.569(12)
I(2)^{2} − Pb(1) − I(1)	87.857(11)
I(2)^{2} − Pb(1) − I(1)^{1}	89.568(12)
I(2) − Pb(1) − I(1)^{1}	87.858(11)
I(2) − Pb(1) − I(2)^{2}	176.395(19)
I(3) − Pb(1) − I(1)	176.239(9)
I(3)^{3} − Pb(1) − I(1)^{1}	176.239(9)
I(3)^{3} − Pb(1) − I(1)	89.499(7)
I(3) − Pb(1) − I(1)^{1}	89.499(7)
I(3)^{3} − Pb(1) − I(2)^{2}	86.998(7)
I(3) − Pb(1) − I(2)^{2}	95.505(7)
I(3) − Pb(1) − I(2)	86.997(7)
I(3)^{3} − Pb(1) − I(2)	95.505(7)
I(3) − Pb(1) − I(3)^{3}	92.356(7)
Pb(1)^{1} − I(1) − Pb(1)	91.160(15)
Pb(1)^{4} − I(2) − Pb(1)	176.397(19)
Pb(1)^{5} − I(3) − Pb(1)	180.0

^{1}1–X, 1–Y, 2–Z; ^{2}X, Y, 1+Z; ^{3}1–X, Y, 2–Z; ^{4}X, Y, –1+Z; ^{5}3/2–X, 3/2–Y, 2–Z

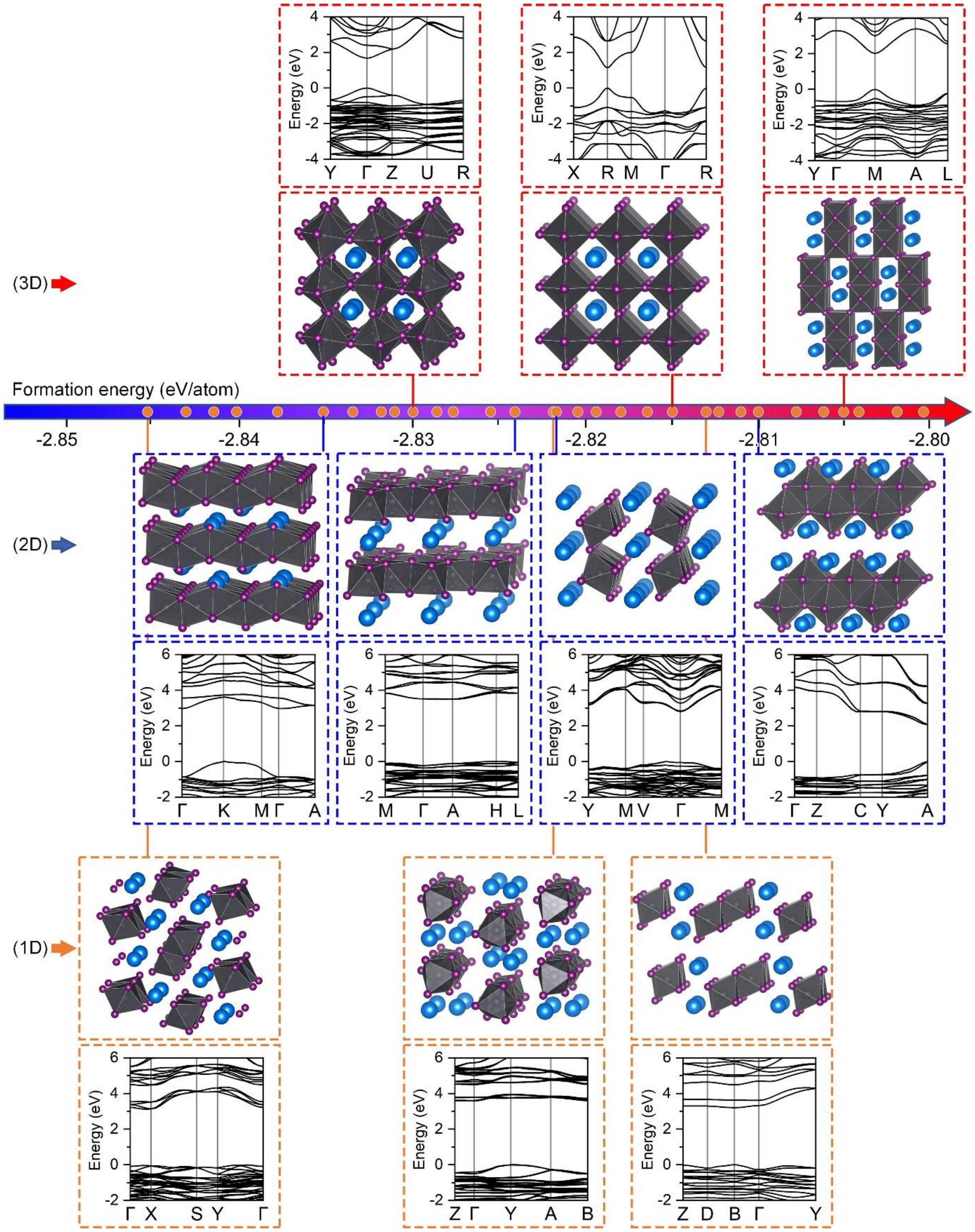

Fig. S1 Crystal structures, formation energies, and band structures of typical polymorphs of $CsPbI_3$ predicted by the structure search using the CALYPSO code

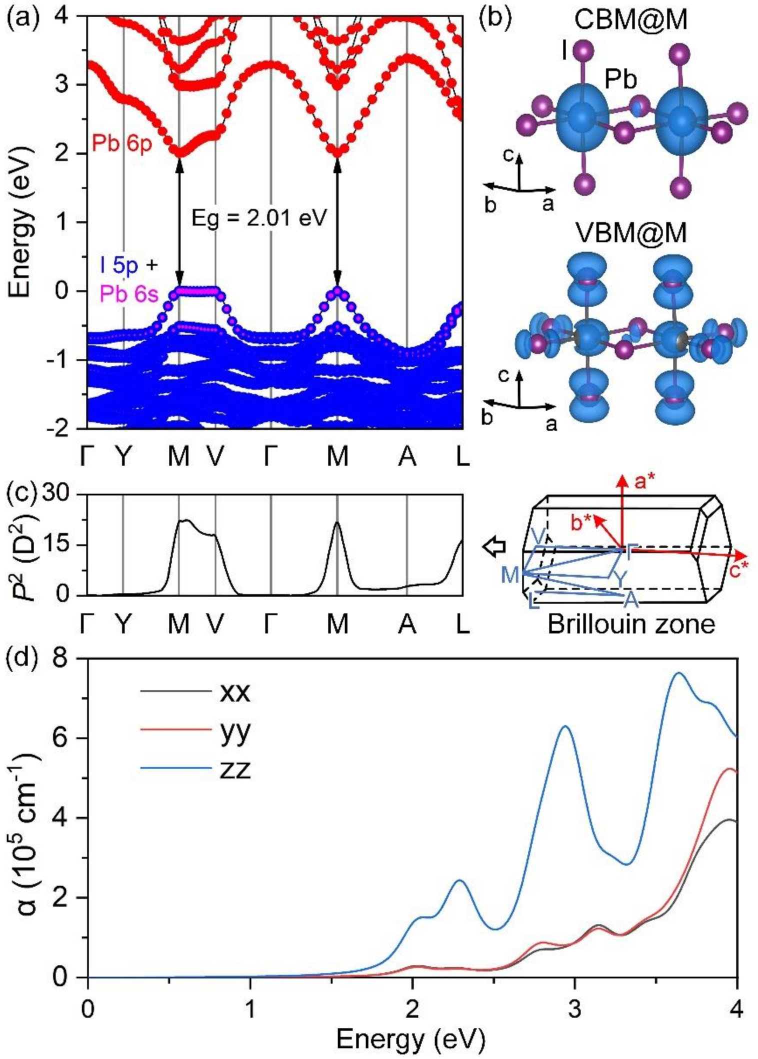

Fig. S2 Electronic properties of the newly-predicted honeycomb-like $CsPbI_3$. (a) Calculated band structure along the *k*-path shown in the inset Brillouin zone. (b) Isosurface plots of charge density corresponding to the conduction band minimum (CBM) and valence band maximum (VBM) at the M point. (c) Calculated transition matrix elements (unit: Debye^2) along the *k*-path shown in the inset Brillouin zone. (d) Calculated optical absorption coefficients as a function of the photon energy

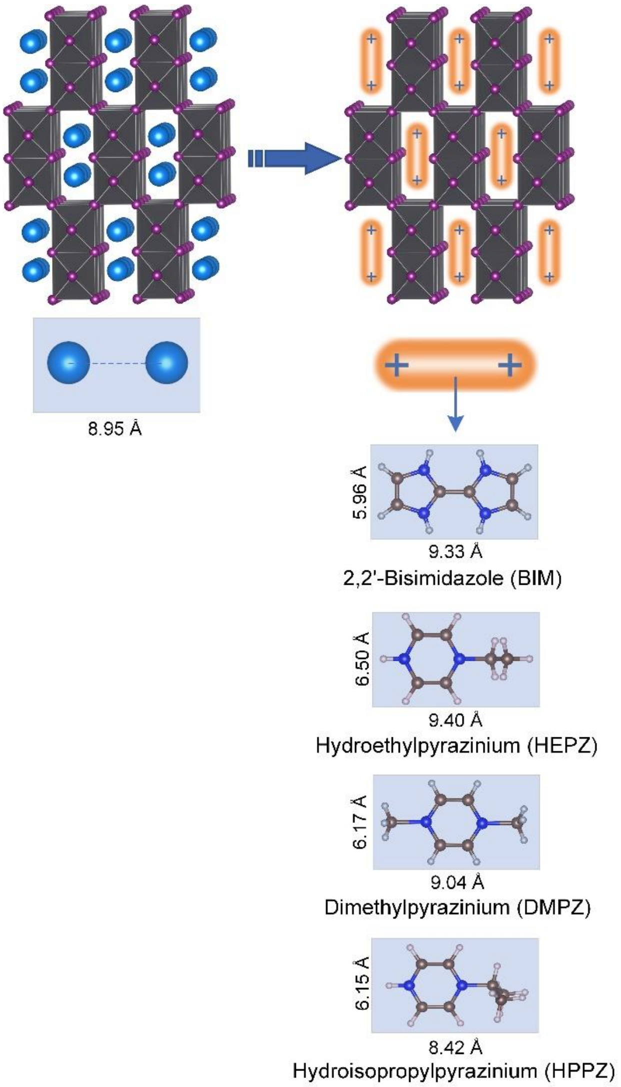

Fig. S3 Schematic of replacing Cs−Cs dimers with organic dications. The yellow capsules represent the organic cations, Bisimidazole (BIM) is found to have suitable size (appx. 9 Å × 6 Å) for honeycomb-like frameworks

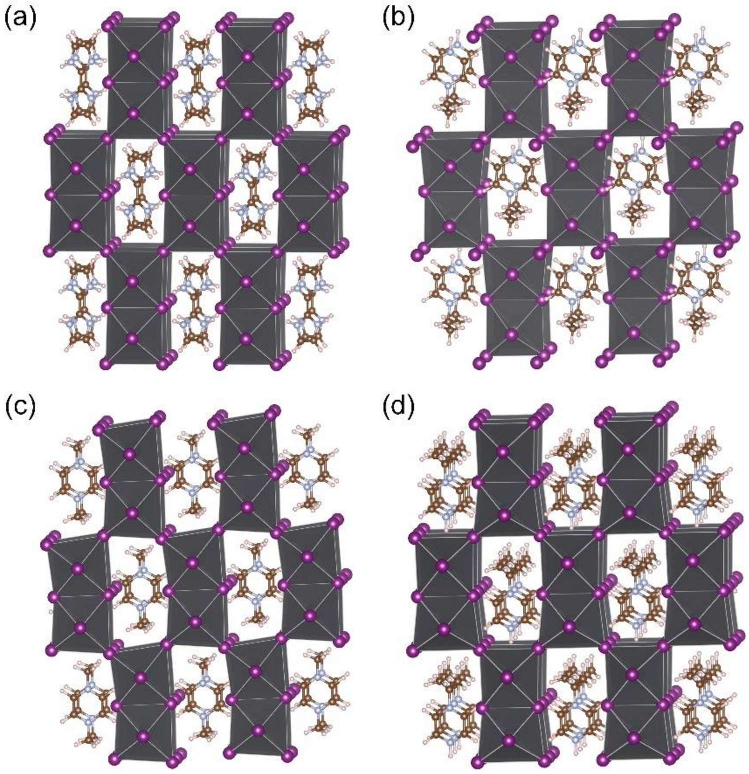

Fig. S4 DFT-relaxed structures of (a) $BIMPb_2I_6$, (b) $HEPZPb_2I_6$, (c) $DMPZPb_2I_6$, and (d) $HPPZPb_2I_6$. Note that BIM cations can perfectly insert into honeycomb-like frameworks with little distortion

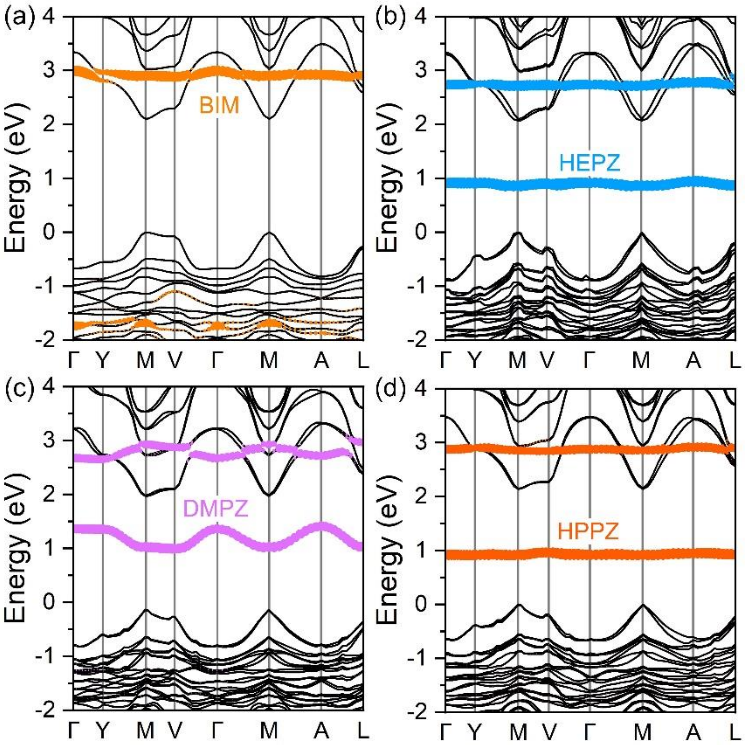

Fig. S5 Calculated band structures of (a)BIMPb_2I_6, (b) BPYPb_2I_6, (c) DMPZPb_2I_6, and (d) HPPZPb_2I_6. Note that except for BIMPb_2I_6, the conduction bands of BPYPb_2I_6, DMPZPb_2I_6, and (d) HPPZPb_2I_6 are derived from the localized molecular orbitals of the divalent molecular cations

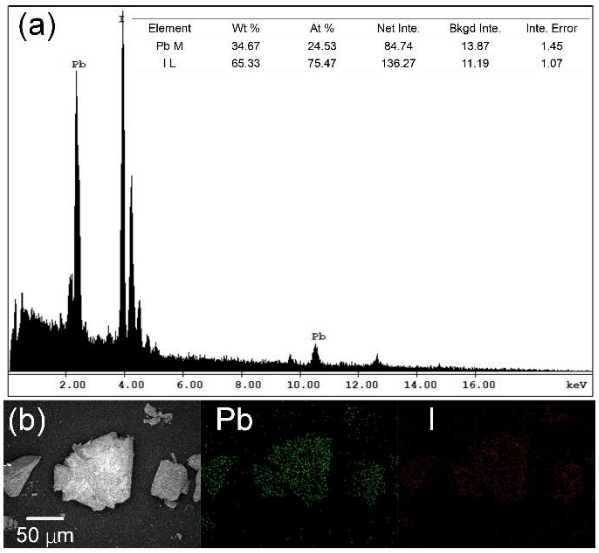

Fig. S6 Elemental analysis by energy dispersive spectrometer (EDS). (a) Atomic ratio of Pb to I is correspond to chemical formula of BIMPb_2I_6. (b) Mapping data indicate both Pb and I are well-distributed in the solid

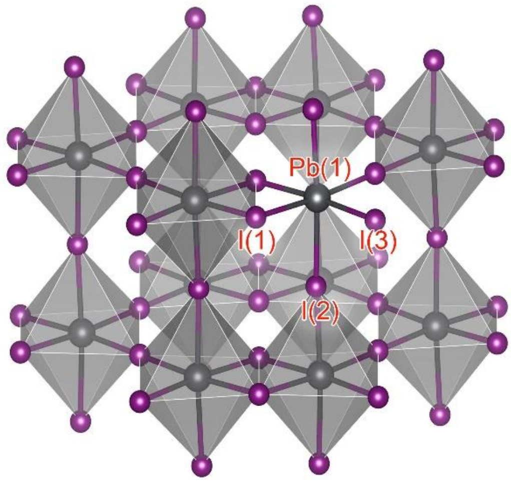

Fig. S7 Combined ball-and-stick or skeletal and shaded polyhedral representations of the inorganic framework of $BIMPb_2I_6$. The elements and positions of asymmetric units are labeled

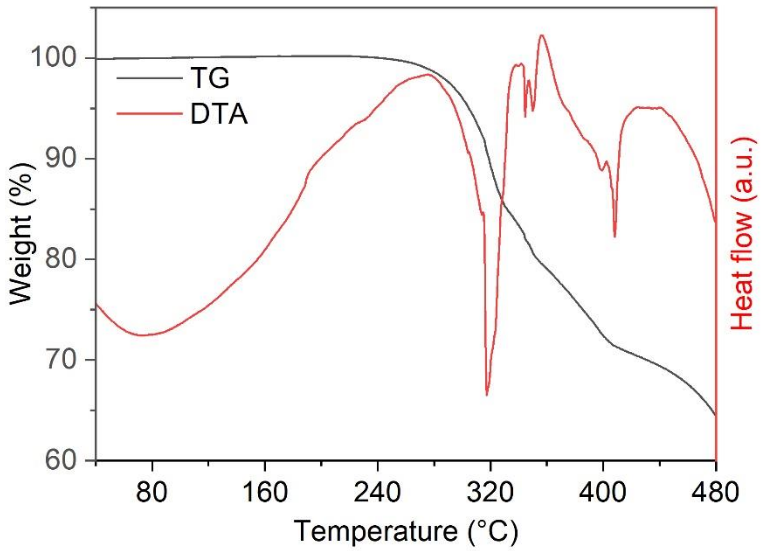

Fig. S8 Thermogravimetric analysis of compound at the temperature range of 60°C–480°C

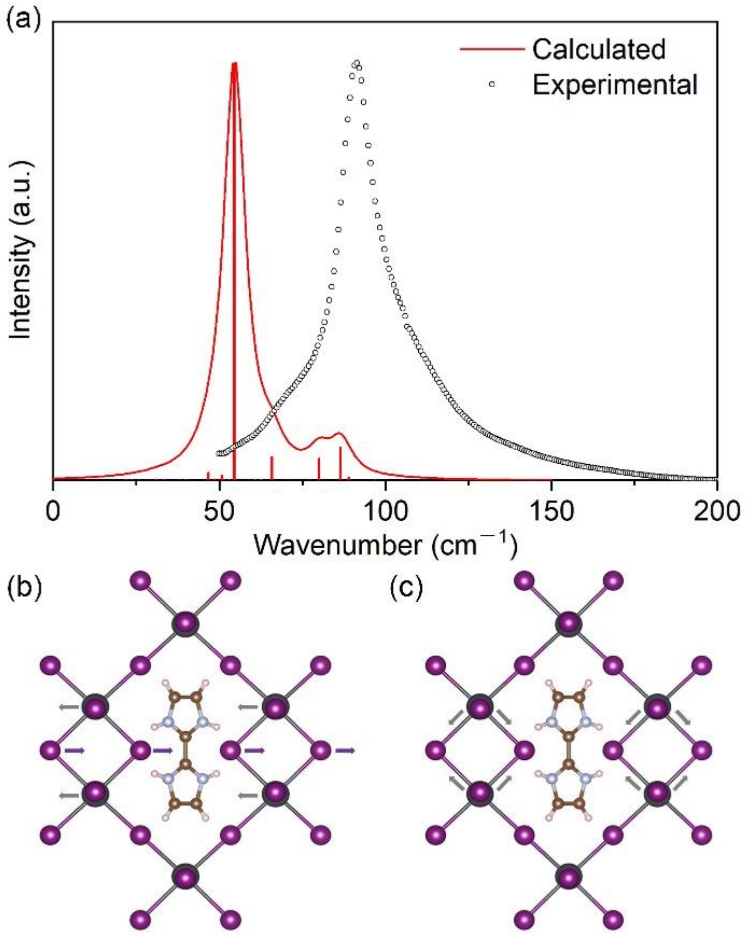

Fig. S9 (a) Experimental resonant and calculated nonresonant Raman signals of $BIMPb_2I_6$ crystals. (b) Bending mode of $BIMPb_2I_6$ corresponding to the signal at 54 cm^{-1}. (c) Stretching mode of $BIMPb_2I_6$ corresponding to the broad peaks centered at around 86 cm^{-1}

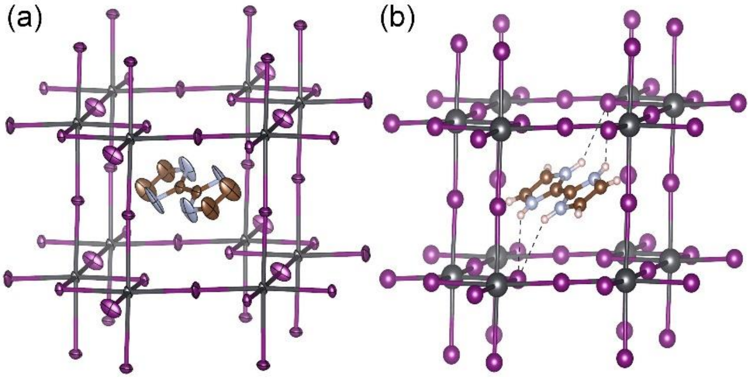

Fig. S10 (a) Thermal ellipsoid models from the SC-XRD data of $BIMPb_2I_6$ at 150 K (ellipsoids at the 90% probability level, H atoms are omitted for clarity). (b) weak H-bonding interaction between BIM molecule and inorganic frameworks (∠N–H–I = 142°)

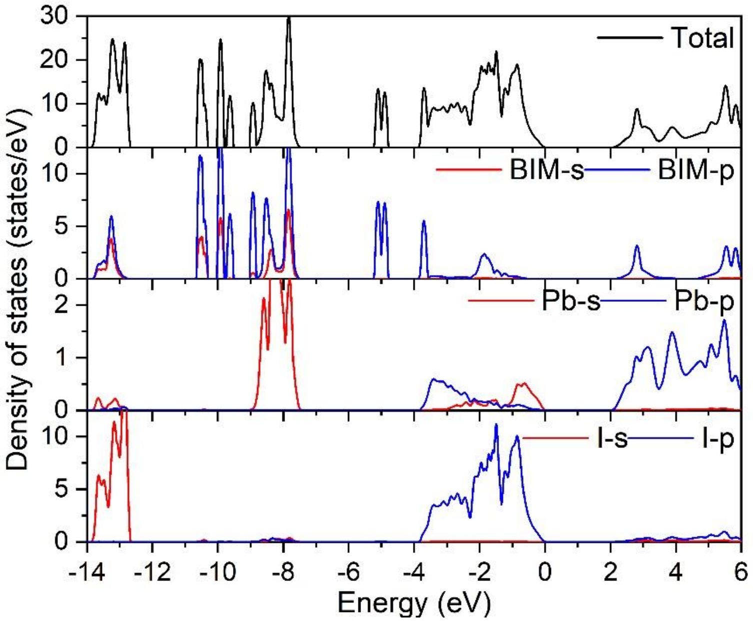

Fig. S11 Calculated total and projected densities of states of $BIMPb_2I_6$

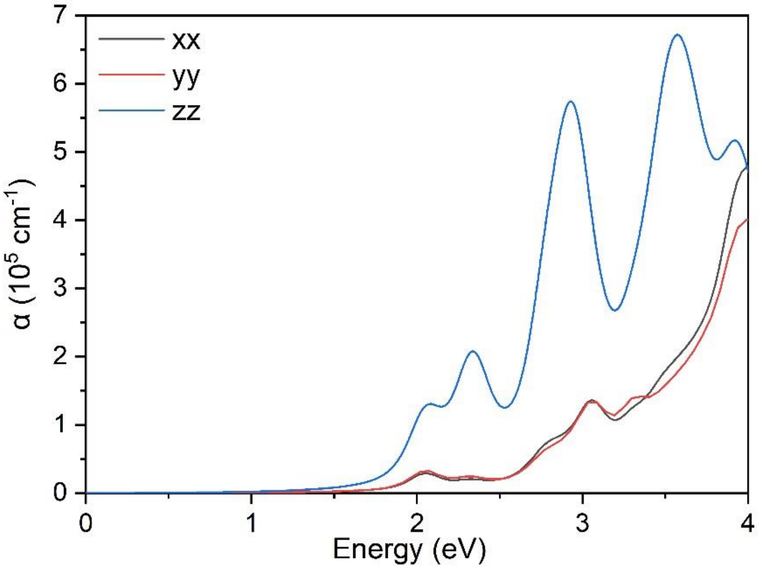

Fig. S12 Calculated optical absorption coefficients of $BIMPb_2I_6$

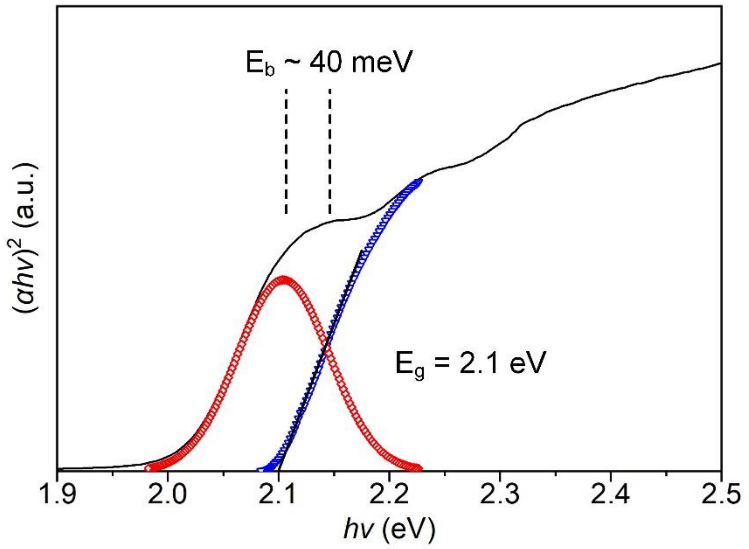

Fig. S13 Experimental and fitted absorption edges of $BIMPb_2I_6$. The exciton binding energy E_b equals to the gap between the exciton peak and the band edge

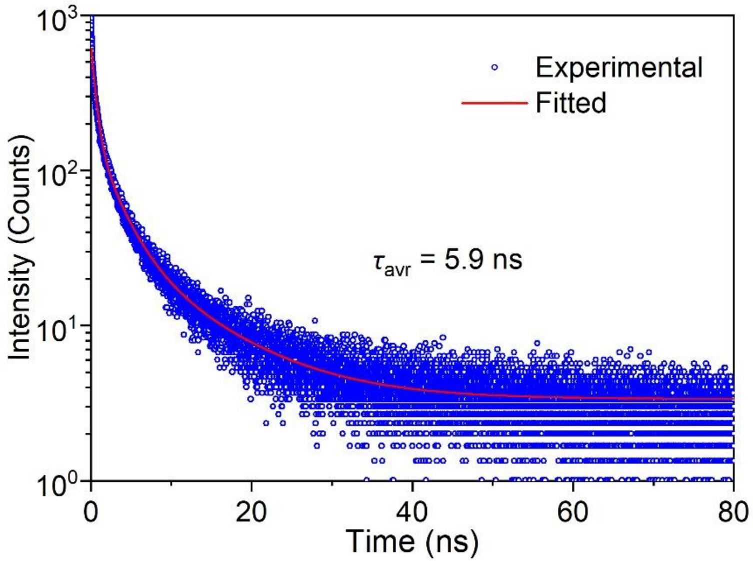

Fig. S14 Time-resolved photoluminance of $BIMPb_2I_6$ monitored at 577 nm (excited by 470 nm light source)
